# From reads to insight: a hitchhiker’s guide to ATAC-seq data analysis

**DOI:** 10.1186/s13059-020-1929-3

**Published:** 2020-02-03

**Authors:** Feng Yan, David R. Powell, David J. Curtis, Nicholas C. Wong

**Affiliations:** 1 Australian Centre for Blood Diseases, Central Clinical School, Monash University, Melbourne, VIC Australia; 2 Monash Bioinformatics Platform, Monash University, Melbourne, VIC Australia; 3 Department of Clinical Haematology, Alfred Health, Melbourne, VIC Australia

## Abstract

Assay of Transposase Accessible Chromatin sequencing (ATAC-seq) is widely used in studying chromatin biology, but a comprehensive review of the analysis tools has not been completed yet. Here, we discuss the major steps in ATAC-seq data analysis, including pre-analysis (quality check and alignment), core analysis (peak calling), and advanced analysis (peak differential analysis and annotation, motif enrichment, footprinting, and nucleosome position analysis). We also review the reconstruction of transcriptional regulatory networks with multiomics data and highlight the current challenges of each step. Finally, we describe the potential of single-cell ATAC-seq and highlight the necessity of developing ATAC-seq specific analysis tools to obtain biologically meaningful insights.

**Electronic supplementary material:**

**Supplementary information** accompanies this paper at 10.1186/s13059-020-1929-3.

## Introduction

Mammalian DNA is highly condensed through three major hierarchical scales; the first is the nucleosome which then wraps into chromatin leading to the third hierarchy, the chromosome [1–6]. Chromatin can dynamically switch between transcriptionally active euchromatin and inactive heterochromatin [7, 8]. All three scales of DNA condensation and their interplay contribute to gene regulation.

Recent gene regulation studies have focused on epigenetics, and through the advances of high-throughput sequencing technologies, various assays have been developed to decipher the epigenetic landscape. These include Assay of Transposase Accessible Chromatin sequencing (ATAC-seq) [9, 10], DNase I hypersensitive sites sequencing (DNase-seq) [11–13] and Formaldehyde-Assisted Isolation of Regulatory Elements sequencing (FAIRE-seq) [14], all of which interrogate chromatin accessibility; Chromatin Immuno-Precipitation sequencing (ChIP-seq) which measures transcription factor (TF) binding [15–17] and histone modifications [18, 19]; and Micrococcal Nuclease sequencing (MNase-seq) which detects nucleosome positioning and occupancy [20, 21]. Detailed procedures of these assays are out of scope of this review and discussed in detail elsewhere [22].

**Fig. 1:**
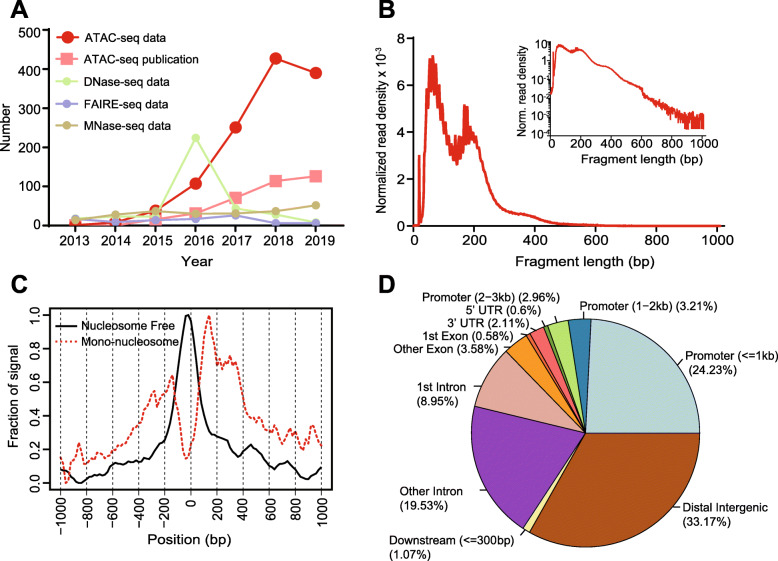
Overview of ATAC-seq datasets increase and sample output for pre-analysis and advanced analysis. **a** The number of ATAC-seq datasets, ATAC-seq publications, DNase-seq datasets, FAIRE-seq datasets, and MNase-seq datasets in PubMed from 1 Jan 2013 to 1 Oct 2019. **b** Typical fragment size distribution plot shows enrichment around 100 and 200 bp, indicating nucleosome-free and mono-nucleosome-bound fragments. **c** Typical TSS enrichment plot shows that nucleosome-free fragments are enriched at TSS, while mono-nucleosome fragments are depleted at TSS but enriched at flanking regions. **d** Typical peak annotation pie chart shows that more than half of the peaks fall into enhancer regions (distal intergenic and intronic regions), and only around 25% of the peaks are in promoter regions. TSS: transcription start site

Among assays designed for detecting chromatin accessibility, ATAC-seq has gained particular popularity since first described in 2013. An exponential increase of curated ATAC-seq datasets and publications indicates its value in a wide spectrum of biological questions (Fig. 1a), such as depicting enhancer landscapes in healthy mammalian tissue and cell types [23–25], studying accessibility changes between normal hematopoiesis and leukemia [26, 27], as well as the chromatin state within schizophrenia patients and the Cancer Genome Atlas (TCGA) pan-cancer cohort [28, 29]. A schematic diagram of this cutting-edge technology in fundamental and translational research is shown in Fig. 3a. Briefly, ATAC-seq incorporates a genetically engineered hyperactive Tn5 transposase that simultaneously cuts open chromatin leaving a 9-bp staggered nick and ligates high-throughput sequencing adapters to these regions. During this process, the nick is repaired, leaving a 9-bp duplication [30, 31]. Paired-end sequencing is then performed to facilitate higher unique alignment rates of these open regions [32].

The hyperactivity of Tn5 transposase makes the ATAC-seq protocol a simple, time-efficient method that requires 500–50,000 cells [9]. The sensitivity and specificity are comparable to DNase-seq but superior to FAIRE-seq where both methods require millions of cells as input material [9]. Because ATAC-seq does not involve rigorous size selection during library preparation, it can also identify nucleosome positions using fragments representing nucleosome monomer and multi-mers [9]. Recently, single-cell ATAC-seq (scATAC-seq) has been described, using fluorescence-activated cell sorting (FACS), microfluidic, and nano-well-based approaches [33–35]. ScATAC-seq can be applied in multiple situations including clinical specimens and developmental biology to study the heterogenous cell populations at single-cell resolution [23, 29].

Despite the simplicity and robustness of ATAC-seq, a major impediment exists as there are few bioinformatic analysis tools developed specifically for ATAC-seq data [32, 36]. Analysis tools used in ChIP-seq and DNase-seq have been applied to ATAC-seq assuming similar data characteristics [37]. However, this assumption has not been evaluated systematically.

**Fig. 2:**
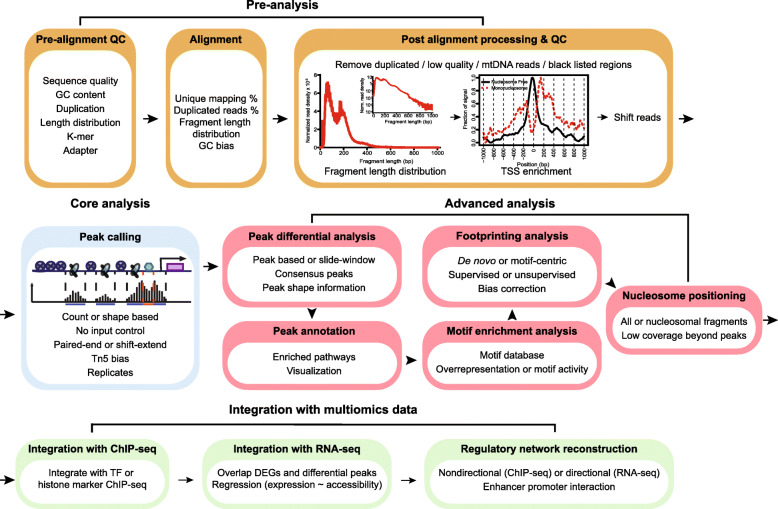
Roadmap of a typical ATAC-seq analysis. Four major steps are listed, including pre-analysis, core analysis, advanced analysis, and integration with multiomics data. Pre-analyses include pre-alignment QC, alignment and post-alignment processing, and QC. Core analysis includes peak calling. Advanced analyses include peak, motif, footprint, and nucleosome analysis. Multiomics data integration includes integration with ChIP-seq and RNA-seq data and regulatory network reconstruction. Text in each box emphasizes the important considerations in each analysis step. We suggest researchers start with FastQC, trimmomatic, and BWA-MEM for pre-analysis, MACS2 for peak calling, csaw for peak differential analysis, ChIPseeker for annotation and visualization, MEME suite for motif detection and enrichment, HMMRATAC for nucleosome detection, HINT-ATAC for footprint analysis, and PCEA for regulatory network reconstruction with RNA-seq. QC: quality check; TSS: transcription start site; TF: transcription factor; DEG: differentially expressed gene

The major focus of this review is to discuss current resources for ATAC-seq analysis. We aim to provide an annotated guide for ATAC-seq data analysis instead of an exhaustive collection of tools. Previous reviews regarding ATAC-seq data analysis have focused mainly on peak callers and modeling regulatory networks [37, 38], but a systematic review covering major parts of ATAC-seq data analysis is urgently needed. This review will cover the four most important steps listed in the flowchart (Fig. 2). These include (1) pre-analysis (quality control (QC) and alignment), (2) core analysis (peak calling), (3) advanced analysis at the level of peaks, motifs, nucleosomes, and TF footprints, and (4) integration with multiomics data to reconstruct regulatory networks. These steps will allow researchers to conduct robust analysis on ATAC-seq data and generate more biological meaningful results. Finally, we will present the challenges and opportunities of ATAC-seq analysis and scATAC-seq.

## Pre-analysis: quality control and alignment

The first step of ATAC-seq analysis involves pre-alignment QC, read alignment to a reference genome, and post-alignment QC and processing (Fig. 2a) [32].

### Pre-alignment quality control

The pre-alignment QC and read alignment steps are standard for most high-throughput sequencing technologies. For example, FastQC [39] can be used to visualize base quality scores, GC content, sequence length distribution, sequence duplication levels, k-mer overrepresentation and contamination of primers and adapters in the sequencing data. An overall high base quality score with a slight drop towards the 3′ end of sequencing reads is acceptable. No obvious deviation from expected GC content and sequence read length should be observed. Moreover, the metrics should be homogeneous among all samples from the same experimental batch and sequencing run.

Currently, due to the ubiquitous use of Illumina’s Nextera library for ATAC-seq, overrepresentation of Nextera sequencing adapters is often observed and should be removed for accurate read alignment. Most adapter removal tools employ different variations of dynamic programming, such as cutadapt [40], AdapterRemoval v2 [41], Skewer [42], and trimmomatic [43] all requiring input of known adapter sequences. For example, using trimmomatic with built-in adapter sequences for Nextera and TruSeq library would be a straightforward step. Low-quality bases can also be eliminated using these tools. From our experience, read trimming tools are generally comparable in performance of efficient removal of low-quality and contaminating adapter sequences.

### Alignment

After read trimming, FastQC can be performed again to check the successful removal of adapter and low-quality bases. Trimmed reads are then mapped to a reference genome. BWA-MEM [44] and Bowtie2 [45] aligners are memory-efficient and fast for short paired-end reads. The soft-clip strategy from both aligners allows the overhang of bases on both ends of reads which can further increase unique mapping rates [46]. We suggest that a unique mapping rate over 80% is typical for a successful ATAC-seq experiment. For mammalian species, the suggested minimum number of mapped reads is 50 million for open chromatin detection and differential analysis, and 200 million for TF footprinting based on empirical and computational estimations [10, 12, 47–49].

### Post-alignment processing and quality control

After sequence alignment, as in most DNA sequencing data, basic metrics of the aligned BAM file, such as unique mapping reads/rates, duplicated read percentages, and fragment size distribution can be collected using Picard [50] and SAMtools [51]. Additionally, reads should be removed if they are improperly paired or of low mapping quality. The mitochondrial genome, which is more accessible due to the lack of chromatin packaging [52], and the ENCODE blacklisted regions [53, 54] often have extremely high read coverage, and should also be discarded [33]. Duplicated reads, which are likely to have arisen as PCR artifacts, should also be removed to significantly improve biological reproducibility [48]. These steps will together improve the power of open chromatin detection and produce fewer false positives.

There are additional ATAC-seq-specific quality metrics that need to be evaluated. Typically, a successful ATAC-seq experiment should generate a fragment size distribution plot with decreasing and periodical peaks corresponding to the nucleosome-free regions (NFR) (< 100 bp) and mono-, di-, and tri-nucleosomes (~ 200, 400, 600 bp, respectively) (Fig. 1b) [9, 55]. Fragments from the NFR are expected to be enriched around the transcription start site (TSS) of genes, while fragments from nucleosome-bound regions are expected to be depleted at TSS with a slight enrichment of flanking regions around TSS (Fig. 1c) [55]. These can be evaluated with the tool ATACseqQC [55]. Lastly, reads should be shifted + 4 bp and − 5 bp for positive and negative strand respectively, to account for the 9-bp duplication created by DNA repair of the nick by Tn5 transposase and achieve base-pair resolution of TF footprint and motif-related analyses [9, 33, 56]. Most aforementioned QC and analysis reports can be integrated using MultiQC [57] for an aggregated, user-friendly, and interactive presentation.

A major consideration for the appropriate tools to choose here is often time to result. Read trimming and alignment can be time consuming, and there is always a trade-off between speed and accuracy. In our experience, the following pipeline performs reasonably well: FastQC➔ trimmomatic➔BWA-MEM➔ATACseqQC, and we would suggest this as a good starting point for processing of ATAC-seq data.

## Core analysis: peak calling

The second major step of ATAC-seq data analysis is to identify accessible regions (also referred to as peaks) and is the basis for advanced analysis. A similar process has been comprehensively reviewed for ChIP-seq [58, 59] and DNase-seq [60]. Currently, MACS2 is the default peak caller of the ENCODE ATAC-seq pipeline. To the best of our knowledge, only one peak caller is specifically developed for ATAC-seq [61]. All others are adopted from ChIP-seq and DNase-seq with the assumption that ATAC-seq peak patterns share the same properties. Thus, we will focus on tools that are currently used in ATAC-seq and provide an overview of potential alternatives (Fig. 4a).

**Fig. 3:**
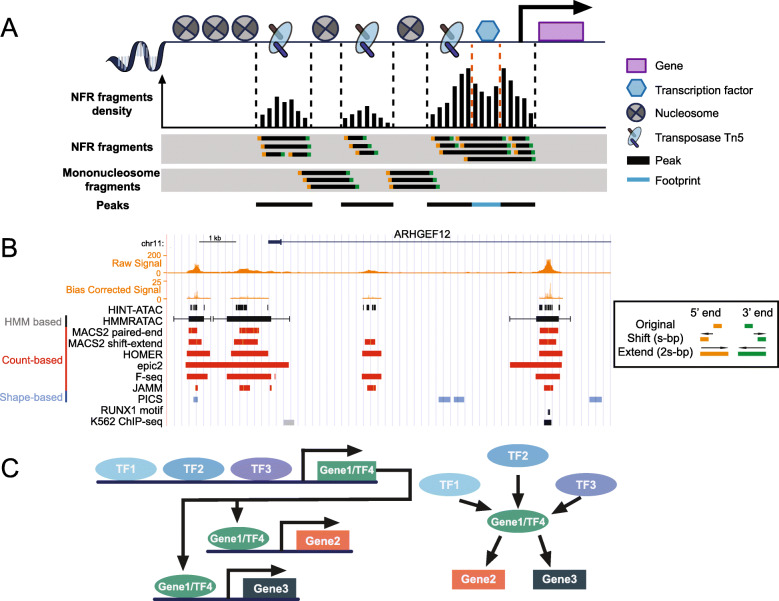
Schematic and real ATAC-seq data from core and advanced analyses. **a** In an ATAC-seq experiment, Tn5 binds and cuts open chromatin and simultaneously ligates adapters. The fragments are sequenced to identify open chromatin regions (black) and footprints (blue). NFR fragments represent the open chromatin, while nucleosome-bound fragments reflect nucleosome positions (gray shaded tracks). **b** Real ATAC-seq data. Signal tracks are generated from BAM file (Raw) and bias corrected by HINT-ATAC (Bias corrected). Peak sets are generated from three types of peak callers, count-based (red), shape-based (blue), and HMM based (black). For MACS2, two strategies (paired-end and shift-extend) are used. For HMMRATAC, the extended ranges at both sides indicate the nucleosomes. The HINT-ATAC track is footprints detected by HINT-ATAC, while the RUNX1 motif track is the footprints matching RUNX1 motif from JASPAR database. The K562 ChIP-seq track is the RUNX1 ChIP-seq from ENCODE, indicating the footprint detection can recapitulate the real TF binding. The right box illustrates the shift-extend approach. First, it shifts both ends s-bp towards outside, and then extend 2s-bp towards inside. **c** Illustration of network reconstruction by ATAC-seq data. The presence of TF can be represented by motifs or footprints detected by aforementioned methods. NFR: nucleosome-free region; TF: transcription factor; HMM: hidden Markov model

Unlike in ChIP-seq, input controls for ATAC-seq, in which Tn5 transposase randomly cleaves protein-free DNA, are often unavailable due to high sequencing costs to obtain comparable coverage. Thus, peak callers which require input controls are impractical for ATAC-seq. Moreover, the direct pile-up of paired-end fragments from ATAC-seq represents both nucleosome-free and nucleosome-bound regions (Fig. 3a). Open chromatin can be detected by piling up short fragments from NFRs or using a shift-extend approach, which tries to count the cutting events smoothed by the extension size (Fig. 3b, right box) [61, 62]. This approach is more generic, as it can be applied to almost all ChIP-seq peak callers and is not affected by the fragment size of data.

Popular peak callers for ATAC-seq can be divided into two major categories: count-based or shaped-based. The count-based peak callers employ different statistical methods to compare read distribution shape in a candidate region to a random background. MACS2 [63], HOMER [64], and SICER/epic2 [65–67] assume Poisson distribution, while ZINBA [68] assumes zero-inflated negative binomial distribution. F-seq [69] and PeakDEck [70] use kernel density estimation to profile fragment distribution. SPP [71] has no assumption on fragment distribution, but uses a sliding window to calculate scores based on fragment counts from up- and downstream flanking windows. One should keep in mind that some tools, such as F-seq and ZINBA, are not actively maintained and should therefore be used with caution. When applying mixture model clustering to biological replicates, JAMM can determine peak width and boundaries more accurately [72]. In general, count-based methods are easier to interpret and widely used.

Shape-based peak callers are not currently used in ATAC-seq, but they utilize read density profile information directly or indirectly and are believed to improve peak calling in ChIP-seq [73]. PICS [74] models fragment positions other than counts and calculate enrichment score for each candidate region. PolyaPeak [75] ranks peaks using statistics describing peak shape. CLC [76] learns a Gaussian filter for peak shape from positive and negative peaks.

Currently, HMMRATAC is the only peak caller that is exclusive for ATAC-seq [61]. It employs a three-state semi-supervised hidden Markov model (HMM) to simultaneously segment the genome into open chromatin regions with high signal, nucleosomal regions with moderate signals, and background regions with low signals, respectively. Although HMMRATAC is computationally more intensive, it performs better than MACS2 and F-seq and provides additional nucleosome position information at the same time.

Other considerations should include whether the peak caller accounts for Tn5 cleavage bias and how it deals with biological replicates. Similar to DNase-seq, the enzymatic cut by Tn5 will introduce bias due to binding preference [30, 31, 77], which is associated with GC content and should be adjusted when calling peaks [22, 56]. Biological replicates can improve reproducibility and reduce false positive peaks. Most tools can be extended to replicates by either pooling raw reads or combining peak sets from individual samples. Replicates can also be integrated using mixture models [72].

Peak tracks generated by these tools can be visualized in Fig. 3b. Count-based tools behave similarly but are quite different from shape-based tools. Furthermore, the underlying sequence features of these peaks were extracted using neural networks and were shown to recapitulate known TF motifs. This confirmed that TFs play an important role in gene regulation through open, accessible chromatin [78, 79]. Fine tuning of parameters is essential for all aforementioned tools [9, 33], as the width of open chromatin varies [32]. Tools that stitch nearby narrow peaks to form broad peaks such as MACS2, HOMER, and SICER/epic2 are also thought to provide more meaningful results. However, to date, there is no comprehensive benchmark study on peak callers for ATAC-seq, and we suggest using actively supported tools, such as MACS2 and HOMER for peak calling, and if computational resources are sufficient, HMMRATAC could be used for ATAC-seq peak calling.

## Advanced analysis

### Peaks

Because by its nature ATAC-seq reveals multiple aspects of transcriptional regulation, the third major step involves interpretation at four different levels: peak, motif, nucleosome, and TF footprint. However, only a few tools are designed specifically for ATAC-seq.

#### Peak differential analysis

**Fig. 4:**
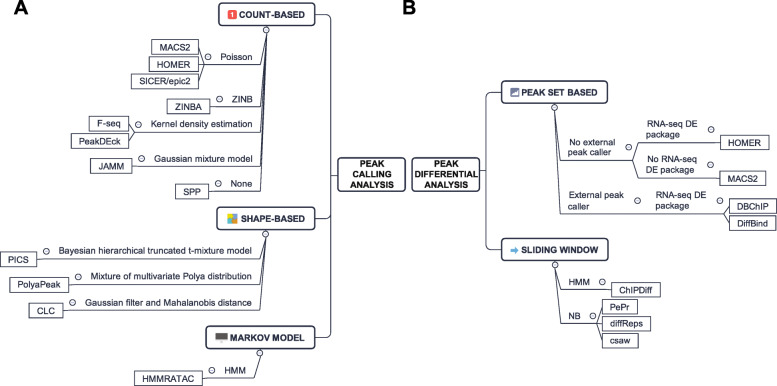
Summary of peak calling and peak differential analysis tools. **a** Peak callers can be divided into count-based, shape-based, and Markov model approaches. They can be further divided by the statistical methods or models used. **b** Peak differential analysis tools can be divided into peak set-based and sliding window approaches. Peak set-based methods are divided based on the usage of external peak caller and RNA-seq DE packages. Sliding window methods are divided based on statistical methods or models used. ZINB: zero-inflated negative binomial; HMM: hidden Markov model; DE: differential expression; NB: negative binomial

Currently, no differential peak analysis tools have been specifically developed for ATAC-seq data analysis. A straightforward approach would be to find the candidate regions (consensus peaks or binned genome), normalize, and count the fragments in these regions and compare with other conditions statistically [80]. This could be achieved manually or using automated tools, such as consensus peak or the sliding window-based tools (Fig. 4b).

Among the consensus peak-based tools, HOMER, DBChIP [81], and DiffBind [82] rely on RNA-seq differential expression (DE) analysis packages, such as edgeR [83], DESeq [84], or DESeq2 [85]. Thus, they all assume a negative binomial (NB) distribution and require biological replicates to estimate dispersion. It has been suggested to call consensus peaks by pooling all samples to reduce false positive differential peaks which is the default behavior for HOMER [86]; however, DBChIP and DiffBind generate consensus peaks by intersection or union operations. Nevertheless, an intersection operation ignores sample or condition specific peaks, and a union operation often shows lower *P* values and more false positives [86].

Sliding window approaches do not require pre-generated peak sets. Instead, they evaluate all windows along the binned genome and tend to yield more false positives and require stringent filtering and false discovery rate (FDR) control. PePr [87] and DiffReps [88] use NB test, G-test, or chi-square test, depending on the availability of replicates. For broader peaks, ChIPDiff [89, 90] employs an HMM to account for correlation between adjacent windows. These three tools are independent of RNA-seq DE analysis packages. In contrast, csaw was developed by extending edgeR framework to binned genomes [91]. The sliding window approach is thought to give more unbiased estimates of read count across the genome but requires rigorous FDR control to properly merge adjacent windows.

Currently, most studies assume that ATAC-seq reads in peak regions follow a NB distribution, as is the case for RNA-seq data. However, no shape-based differential analysis tools exist for ATAC-seq data. The peaks contain not only read count information, but also the distribution shape profile. It is especially important for broad peaks, as broad peaks can contain multiple local maxima, and those shifts can indicate biologically relevant perturbations, which could be detected in sliding window or shape-based methods. Although not systematically studied, we believe incorporating shape information will improve differential peak analysis. Nevertheless, considering replicate handling, external peak caller dependency and backend statistical methods, csaw is worth a first try due to its easily explainable edgeR framework.

#### Peak annotation

After obtaining peak sets, annotation of peaks can associate chromatin accessibility with gene regulation. Normally, peaks are annotated by the nearest genes or regulatory elements. HOMER, ChIPseeker [92], and ChIPpeakAnno [93] are widely used to assign peaks to nearest or overlapping gene, exon, intron, promoter, 5′ untranslated region (UTR), 3′ UTR, and other genomic features. ChIPseeker and ChIPpeakAnno also have abundant visualization functions for interpreting annotation results, such as a pie chart of annotated genomic features (example in Fig. 1d). Typically, peaks from ATAC-seq will represent a mixture of different *cis-*regulatory elements including enhancers and promoters [12]. After obtaining the list of genomic features such as the nearest genes, functional enrichment analysis can also be performed using databases such as Gene Ontology (GO) [94], KEGG [95], and Reactome [96]. In general, peak annotation generates biological and functionally meaningful results for further investigation.

### Motifs

Although peak annotation provides functional interpretation, it does not directly explain the underlying mechanism. Open chromatin can affect transcription through TFs, which facilitate transcription by recognizing and binding to specific sequences on DNA. This sequence is known as a motif and the binding positions are called TF binding sites (TFBS). There are approximately 1600 TFs in human, and more than half have motifs obtained experimentally or computationally [97]. Most TFs require chromatin to be accessible for binding, while certain pioneer TFs can bind to less accessible nucleosomal DNA [98, 99]. TFs regulate transcription through competition with histone or non-histone proteins [100, 101] and cooperation with co-factors [102]. These chromatin accessibility remodeling processes have been reviewed in detail by Klemm, Shipony, and Greenleaf et al. in a recent publication [103]. Thus, understanding motif usage or activity change may help to decipher the underlying regulatory networks, as well as identify key regulators [104]. There are two types of motif or TF-based analysis methods: sequence-based prediction for motif frequency or activity, and footprinting for TF occupancy (discussed in next section).

#### Motif database and scan

In order to exploit motif information, great efforts have been made to compile databases of motif sequences from either experimental methods or computational predictions. Popular databases such as JASPAR [105] contain multiple species and can be easily retrieved using application programming interfaces (APIs) or Bioconductor packages [106, 107]. To name a few databases, CIS-BP [108] and TRANSFAC [109] contain eukaryotic TF motifs, HOCOMOCO [110] focuses on human and mouse, and RegulonDB [111] is specifically for *E. coli*. However, there is no central database, which contains comprehensive and consistent motif information, and the discrepancies can arise from differences of original ChIP-seq experiments and the software used to perform de novo motif discovery.

The motif information is mainly stored in text format, for example, as a position weight matrix (PWM). HOMER and Bioconductor packages TFBSTools [112] and motifmatchr [113] are able to search given nucleotide sequences for putative TFBSs using PWM. PWMScan [114] provides a web server for fast motif scanning using a Bowtie indexed genome. Another widely used tool is MEME suite [115, 116], which includes FIMO [117] to search for individual motifs, MAST [118] for aggregating search results from multiple motifs, and MCAST [119] to infer regulatory modules formed by multiple motifs. These tools generate a list of putative TFBSs based on statistical matching. Among them, MEME suite and PWMScan are more accessible owing to their web application interfaces.

#### Motif enrichment and activity analysis

Based on aforementioned motif search tools, the position and frequency of motifs in each peak region can be obtained and compared to a random background or another condition. HOMER uses the hypergeometric test, while MEME-AME [120] uses the rank-sum test to compare motif frequencies within peaks. MEME-CentriMo [121] further identifies motifs enriched near peak centers. DAStk [62] generates a MD score (motif displacement score) [122]. This is achieved by calculating the ratio of motif occurrence within a small window (150 bp) to a large radius (1500 bp) from each peak center. The MD score can also be compared across different conditions with a *Z*-test. These methods employ different statistical tests to compare the motif frequency in peaks and the background regions.

Apart from an overrepresentation test, accessibility at each putative TFBS is assumed to associate with TF activity and can be measured by fragment counts. ChromVAR [56] calculates accessibility deviation across multiple conditions for each motif using a *Z*-score and is adjusted for known technical bias (GC bias, average accessibility and fraction of reads in peaks). It is specifically designed for scATAC-seq data with a large number of cells that could be considered as replicates. However, its performance in bulk ATAC-seq has not been evaluated yet. DiffTF generates a distribution of accessibility fold changes for all TFBSs, adjusted for GC content for each motif and is then compared to a permutated null background to evaluate significance [123, 124]. In summary, MEME-CentriMo is a widely used web application that produces a visual report, while chromVAR can be an alternative in scATAC-seq.

All tools mentioned so far predict putative TFBSs indirectly from sequences found within peak regions. Such TFBSs can contain a significant fraction of false positives and are likely to be incomplete and confounded. This is because not all TFs have identified motifs and TFs from the same family can share very similar motifs [125]. Moreover, the predicted enrichment or activity change could have negligible biological meaning which hampers the interpretation of the sequence-based motif analysis results.

### Footprints

Another way to decipher the TF regulation is to use footprints. A footprint in ATAC-seq refers to a pattern where an active TF binds to DNA and prevents Tn5 cleavage within the binding site. This leaves a relative depletion within the open chromatin region (Fig. 3a) [47, 126, 127]. Thus, footprints of actively bound TFs can be used to reconstruct a regulatory network specifically for certain samples.

However, there are hurdles for ATAC-seq footprinting analysis. First, it is important to shift the raw reads in the pre-processing step to account for the 9-bp duplication for accurate footprint detection [9, 33]. Second, due to binding preference of Tn5 [32, 128] and the weak signal of transient TF binding [129], footprint detection is both experimentally and computationally difficult [130]. Great efforts have been made in DNase-seq footprinting, which faces similar challenges except for the difference in enzymatic bias. However, only a few footprinting tools have been tested on ATAC-seq and no systematic evaluation has been performed [48, 131, 132].

Footprinting analysis tools mainly fall into two categories: de novo and motif-centric methods. De novo methods predict all footprint sites across peaks, according to features of a typical footprint pattern (peak-dip-peak). Then these putative footprint sites are used to match known motifs or identify novel motifs. Instead, motif-centric methods require the input of a priori TFBSs and discriminate these sites as bound or unbound using supervised or unsupervised methods (Table 1).

**Table 1:** Summary of footprinting tools, including software category, programming language, algorithm or statistical method, bias correction for DNase-seq or ATAC-seq, and output statistics. In addition, the second last column exemplifies the application of tools in ATAC-seq data

Tool	Category	Language	Algorithm	Bias correction?	Statistics	Used for ATAC in literature?	Reference
Neph	De novo	C++	Slide window	N	Footprint occupancy score (FOS)	N	[47]
HINT		Python	HMM	N	Probability	N	[133]
HINT-BC		Python	HMM	Y (DNase-seq)	Probability	Y [48]	[130]
HINT-ATAC		Python	HMM	Y (ATAC-seq)	Probability	Y [134]	[134]
Boyle		NA	HMM	N	Probability	N	[135]
Wellington		Python	Binomial test	N (visualize bias)	*P* value, FDR	Y [48]	[136]
Wellington-bootstrap		Python	Bootstrap DE analysis	N (visualize bias)	*P* value, FDR	Y [48]	[137]
DNase2TF		R	Binomial test, iteratively merge	Y (DNase-seq)	FDR	Y [134]	[129]
CENTIPEDE	Motif-centric	R	Bayesian mixture model, unsupervised	N	Posterior probability	Y [138]	[139]
msCentipede		Python and Cython	Bayesian multiscale model, unsupervised	Y (can extend to ATAC-seq)	Posterior probability	Y [140]	[140]
Romulus		R	Bayesian mixture model, unsupervised	N	Posterior probability	N	[141]
PIQ		R	Gaussian process model, unsupervised	N	Probability of binding times local chromatin accessibility	Y [134]	[147]
BinDNase		R	Logistic regression, supervised	N	Probability	N	[142]
MILLIPEDE		R	Logistic regression, supervised	N (robust to bias)	Probability	N	[143]
DeFCoM		Python	SVM, supervised	N	Ranking	Y [131, 134]	[131]
BPAC		Python	Random forest, supervised	N	Probability	N	[144]
BaGFoot		R	Differential motif activity	Y	*P* value	Y [132]	[132]

*FDR* false discovery rate, *HMM* hidden Markov model, *SVM* support vector machine

#### De novo tools

For de novo methods, it is important to mathematically define what is a footprint and denoise the footprint pattern from Tn5 cleavage bias [128, 134]. Boyle et al. [135] proposed an HMM using normalized and smoothed fragment counts at each base to detect different states such as footprint, flanking, and background. HINT, HINT-BC (bias correction for DNase-seq), and recent HINT-ATAC also employ HMM, but only HINT-ATAC corrects for strand-specific Tn5 cleavage bias (Fig. 3b) [130, 133, 134]. An example was shown in Fig. 3b, where footprints detected by HINT-ATAC in a leukemia sample were also validated in a K562 cell line with RUNX1 ChIP-seq. Because these HMM-based methods require supervised training using manually annotated genomic regions, their generalizability in larger datasets needs to be further evaluated. Wellington and Wellington-bootstrap [136, 137] compare the number of Tn5 cuts in flanking and candidate footprint region to find the local minima. Bias correction is not considered by Neph’s method, Boyle’s method, HINT, and Wellington, while DNase2TF and HINT-BC do account for bias correction for DNase-seq [47, 129]. Parameter tuning is a critical consideration and will affect the resultant calls. An optimized pipeline using HINT and Wellington has been described, which evaluates results using area under curve (AUC) analysis considering ChIP-seq binding sites as true positive [48]. In summary, only HINT-ATAC currently handles ATAC-seq-specific bias.

#### Motif-centric tools

Motif-centric methods focus on a priori TFBSs and consider TF-specific footprint profiles compared to de novo methods. The challenge is to avoid ascertainment bias where TFs with high-quality motifs are enriched.

The unsupervised motif-centric methods classify putative TFBSs as bound or unbound, based on features extracted from genomic regions, e.g., distance to TSS, PWM match score, and sequence conservation score [145, 146], as well as from sequencing reads, e.g., read number and shape distribution around the putative TFBSs [139–141, 147]. Among them, CENTIPEDE models read distribution with a multinomial model, and its performance is sensitive to parameters in a TF and cell-type-specific way [133, 139, 143], whereas msCentipede and Romulus account for these heterogeneous footprint profiles [140, 141]. Additionally, msCentipde can model Tn5 bias and Romulus improves performance for low depth data and low-quality motifs. PIQ [147] uses a Gaussian process to model read distribution and can further increase robustness when replicates are provided. The accuracy of unsupervised tools relies heavily on feature selection and construction, thus feature engineering and selection techniques, such as one-hot encoding, binning, and clustering, can be attempted to further improve performance.

In contrast, supervised motif-centric tools require high-quality ChIP-seq to annotate true TFBSs as training data. MILLIPEDE and BinDNase both use logistic regression [142, 143], while DeFCoM uses support vector machine (SVM) and BPAC uses a random forest classifier [131, 144]. Specifically, BinDNase trains a model for each TF separately to account for the TF-specific footprint pattern. The SVM approach used in DeFCoM is more robust to outliers compared to logistic regression [131]. Additionally, DeFCoM was tested on ATAC-seq data and showed slightly decreased performance compared to in DNase-seq with twice the read number. For all supervised tools, performance decreases in cross-TF/cell-type validation, due to variable footprint patterns [142]. This could hamper their application in rare cell populations or heterogeneous cancer samples. An ensemble of larger and more diverse training data was shown to improve footprinting performance [144], and we would also expect ensemble learning to be beneficial, where multiple learners are trained to predict collectively. Moreover, all these tools are trained using DNase-seq data, thus they should be retrained using ATAC-seq data to account for intrinsic bias of different data. In general, modeling TF and cell-type-specific footprint patterns remains difficult due to their substantial variability.

If global TF footprint pattern changes between conditions is of interest, BaGFoot [132] can be employed. It calculates footprint depth and flanking accessibility for all TFs after sequence depth normalization and bias correction. This method is robust to assay type (DNase-seq or ATAC-seq), peak caller, and bias correction methods [132].

#### Comments on footprinting analysis

There are several caveats for footprinting analysis. First, supervised motif-centric footprinting tools generally outperform unsupervised counterparts and de novo methods, with the trade-off of less generalizability [130, 131]. They have been trained using ChIP-seq and DNase-seq data from specific TFs in specific cell types. Therefore, their context may not be generalizable and applicable to ATAC-seq. Moreover, training data is not always available from the sample of interest, and cross-TF/cell-type prediction should be conducted with caution [131, 144]. Generalizability of these tools to ATAC-seq still requires extensive evaluation. Second, bias correction is important in both DNase-seq and ATAC-seq footprint detection. Recently, the Tn5 preferential motif has been identified and shown to confound some C2H2 zinc finger TFs [128]. Third, there is not a general guideline for minimal ATAC-seq sequencing depth in order to achieve effective footprinting. Although more than 200 million reads per sample are recommended, DeFCoM has been described to work comparably well with fewer sequencing reads [10, 48, 131]. With increasing depth, the improvement of footprinting varies between TFs and cell types due to different binding affinity and turnover [131]. However, saturation analysis is needed to provide reasonable suggestion for experimental design regarding sequencing depth per sample. Fourth, de novo methods still have the advantage for low-quality and novel motifs. Although the evaluation of footprint methods is inconsistent across different studies because of the analysis tools chosen, parameter setting, and evaluation metrics, we believe that HINT-ATAC can be a decent option due to its ATAC-seq-specific bias correction [130, 131]. Additionally, researchers could combine the results from multiple tools to obtain highly reliable footprints. Nevertheless, footprinting analysis in ATAC-seq is useful to understand TF regulation and further reconstruct cell-specific regulatory networks, and therefore requires extensive benchmarking for software comparison and development within specific contexts.

### Nucleosome positioning

The nucleosome consists of a histone octamer complexed with approximately 147 bp of DNA (Fig. 3a) and affects TF binding by altering chromatin accessibility [2, 103, 148]. In a standard ATAC-seq library, longer fragments correspond to nucleosome-associated regions (Fig. 3a) [9]. There are analysis tools developed to detect regions where these nucleosomal fragments are enriched. However, Schep et al. showed that nucleosome detection in ATAC-seq was more difficult than in MNase-seq data, due to the deceased read coverage beyond the open chromatin regions [149].

Software developed for MNase-seq, such as DANPOS2, PuFFIN, iNPS, and NucTools, can be applied to ATAC-seq data after filtering for nucleosome-associated fragments [149–153], while NucleoATAC and HMMRATAC are developed specifically for ATAC-seq. NucleoATAC outperformed DANPOS2 by devising a signal score for each base by cross-correlating positional signals with V-plots. A V-plot is a dot-plot to visualize fragment size and midpoint position and is conserved across species [149, 154, 155]. The signal score is normalized and smoothed, and local maxima are found by log-likelihood. HMMRATAC can simultaneously detect open chromatin and nucleosome-associated regions as discussed before (Fig. 3b) [61]. Moreover, DANPOS2 and NucTools can detect the nucleosome occupancy change and position shift between conditions [150, 151]. INPS incorporates a wavelet denoising method while PuFFIN sums up nucleosomal fragment distributions weighted by fragment sizes to identify nucleosomes [152, 153, 156].

However, all these tools suffer from the same underlying drawbacks of a typical ATAC-seq experiment, which is low coverage beyond open chromatin. In the future, new experimental protocols coupled with bioinformatic methods for ATAC-seq will be required to more efficiently and precisely capture nucleosome occupancy. Here, we believe HMMRATAC and NucleoATAC are two useful and specific tools for ATAC-seq nucleosome detection.

## Integration with multiomics data to reconstruct regulatory networks

Along with the specified requirements for ATAC-seq data analysis we have articulated so far, the integration of ATAC-seq with other high-throughput sequencing technologies such as RNA-seq and ChIP-seq is gaining increasing interest to understand gene regulation.

### Integration with ChIP-seq

Because open chromatin is the pre-requisite for most TFs to bind, ATAC-seq peaks generally overlap with TF ChIP-seq peaks but are often broader. Thus, TF ChIP-seq and ATAC-seq can mutually validate the quality and reliability of each other within the same experimental system [157]. Unique peaks in TF ChIP-seq could indicate pioneer TFs that bind to closed chromatin, which then recruit chromatin remodellers or other TFs and initiate transcription [98, 103]. Analysis based on putative TFBS, such as motif enrichment and footprint detection, can be further improved by incorporating true TF ChIP-seq peaks to reduce false positives [54]. ATAC-seq can also be integrated with histone marker ChIP-seq and is found to positively correlate with active chromatin makers (H3K4me3, H3K4me1, H3K27ac, etc.) and negatively correlate with inactive chromatin markers (H3K27me3) [9, 157, 158]. In conclusion, integrating ChIP-seq and ATAC-seq helps to understand TF and histone facilitated chromatin accessibility changes. We foresee ATAC-seq to be a pioneer assay before specific TF ChIP-seq, due to the ease of the protocol and less sample requirement.

### Integration with RNA-seq

Researchers are also interested in qualitatively or quantitatively associating changes in chromatin accessibility with changes in gene expression by RNA-seq. Intuitively, researchers can discover whether DE genes also have significantly differential chromatin accessibility surrounding the respective TSS [159]. Moreover, DE genes can be inferred to be regulated by TF associated with specific motifs or footprints in open chromatin. At the single cell level, Litzenburger et al. attempted to combine scRNA-seq and scATAC-seq to identify the target genes whose expression varies when GATA binding site accessibility changes [160]. Cao et al. used a LASSO regression model to identify distal peaks which account for the target gene expression change [161]. Coupled clustering combining scATAC-seq and scRNA-seq was shown to improve accuracy in subpopulation detection [162]. Integration of ATAC-seq with RNA-seq aids to decipher gene regulation and cellular heterogeneity.

### Reconstruction of regulatory networks

While ATAC-seq can simultaneously detect hundreds of TF motif occurrences or footprints, it is possible to reconstruct cell-specific regulatory networks by linking footprints/motifs with downstream genes. Similar approaches have been demonstrated in DNase-seq (Fig. 3c) [104, 163]. However, previous attempts in DNase-seq have been restricted to promoter regions and only investigate TF-TF regulation [104]. Peaks within promoters only constitute a small proportion of all ATAC-seq peaks, while the majority are found in distal enhancers reducing the power to infer regulatory networks [9]. Enhancers can be very distant in a linear genome but spatially proximal (in 3D) to their target genes. This leads to the difficulty of predicting direct target genes of enhancers. Many studies have considered distal peaks as enhancers and linked them to the closest gene akin to a promoter analysis [164–166]. With scATAC-seq, Pliner et al. proposed Cicero, which accurately recapitulates co-accessible peaks and links enhancers and promoters to the same target gene. This method has been validated by orthogonal methods [167]. While it has been demonstrated to work in scATAC-seq, it is unclear if this method is applicable to bulk ATAC-seq with much smaller sample sizes. Nevertheless, Cicero is a forerunner in connecting distal enhancers to gene regulation using ATAC-seq.

Although it is possible to reconstruct undirected TF-gene regulatory networks with ATAC-seq alone, the directional regulation can be further inferred as activation or repression when RNA-seq is integrated. Duren et al. proposed a model with paired gene expression and chromatin accessibility (PECA) data to predict the target gene expression as a function of TF expression, chromatin remodeller expression and chromatin accessibility [168]. Miraldi et al. used ATAC-seq-derived binary TF-gene interactions as prior networks, to further refine regulatory networks inferred from RNA-seq data [166]. Berest et al. classified TFs to be activators or repressors based on correlation of TF expression and accessibility at TFBSs across the whole genome [124] with the assumption that accessibility, similar to histone markers, positively correlates with TF expression for activators and negatively for repressors [124, 169]. This method only allows classification in a global manner.

In order to further improve network reconstruction, publicly available ChIP-seq datasets can be integrated to improve the accuracy of footprinting. Incorporating known enhancer-promoter interactions from chromatin conformation data would also be helpful. With the surge of deep learning, it would require more work on feature construction and selection in order to build effective algorithms to predict transcriptional regulatory networks. In summary, integrating ATAC-seq with multiomics data yields biologically meaningful results, which can uncover underlying mechanisms of gene regulation.

## Pipelines for ATAC-seq data

There is growing need for integrated pipelines to process ATAC-seq data. Several have been developed but have different focus for downstream analysis by stitching together previously discussed tools.

To name a few, esATAC [170] and CIPHER [171] focus on peak annotation, while GUAVA [172], a graphic user interface (GUI) tool, focuses on differential peak detection as well as functional annotation. ATAC2GRN [48] is another pipeline specifically optimized for footprinting.

These pipelines will provide a helpful and convenient entry for researchers with minimal programming skills to explore ATAC-seq data. However, a general problem for these pipelines is the lack of flexibility for parameter tuning. Most parameters are hard coded empirically because the combination of them increases exponentially with the number of tools, which makes pipelines difficult to modify for any given context. Overall, a pipeline with visualization and user interface will be more appropriate for nonprogrammers to explore the data.

## Single-cell ATAC-seq

Enabled by microfluidic, nano-well, and combinatorial indexing technologies, scATAC-seq is now able to measure the chromatin accessibility for thousands of cells with easy protocol at a low cost [33–35]. The chromatin accessibility at each base will be binary and the scATAC-seq data will be sparse because in diploid organisms, there are only two copies of DNA. This is a challenge in analyzing scATAC-seq data. Despite the analyses listed for bulk ATAC-seq, another important analysis for single-cell is clustering. A recent benchmarking study from *Chen* et al. about clustering methods in scATAC-seq showed that SnapATAC, *Cusanovich2018* and cisTopic outperformed other methods [23, 173–175]. These three methods are featured by workflows combining window-based genome binning, binarization of the accessibility, coverage bias correction, and dimension reduction using principle component analysis, which specifically handle the sparse scATAC-seq data [175]. This study provides a useful insight for future scATAC-seq software development.

New techniques such as scNMT-seq, sci-CAR, and Pi-ATAC were recently developed to measure chromatin accessibility, transcriptome, and proteome simultaneously from exactly the same cell [161, 176, 177]. Data from these experiments could help to deduce the complex interplay between the epigenome, transcriptome, and proteome and help us to understand why different cells behave distinctively. With the advantages of single-cell analysis are clear, there are challenges. Cost and time-efficient single-cell techniques as well as bioinformatic tools remain an area of active research and development.

## Future perspectives and concluding remarks

ATAC-seq has developed rapidly over recent years and has become a method of choice to investigate chromatin accessibility. There are now optimized protocols that work with single cells, blood samples, and frozen tissue with improved signal-to-noise ratio [26, 33–35, 178]. Despite the progress in protocols, the advancement in bioinformatics analysis tools is slow, with no comprehensive analytical pipeline defined. This imposes a current and ongoing hurdle in the interpretation of ATAC-seq results.

In this review, we have systematically discussed all major steps in an ATAC-seq analysis pipeline for the reader to consider, starting with raw sequencing reads to the endpoint of biological meaningful interpretation. Here, we offer a guide of available tools and suggested steps of analysis to consider to facilitate proper biological interpretation of ATAC-seq data. The alignment and QC steps are similar to RNA-seq and ChIP-seq. As for the peak calling, most ChIP-seq derived tools are compatible with ATAC-seq data. However, a comprehensive benchmarking would help to select appropriate tools and to guide future development of ATAC-seq-specific peak callers. There is growing evidence that improvement or parametrization of current tools can be applied to fit ATAC-seq data.

For downstream interpretation, differential peak analysis can give an overview of the changes of chromatin accessibility. Nevertheless, these changes can arise from both read numbers and the shapes of peaks and can be detected by count-based or sliding window approaches. The performance of these two approaches still requires further evaluation in ATAC-seq and could be specific to particular contexts. In order to infer biological function and related TFs, peak annotation and motif enrichment analysis is a good first pass analysis for initial insight.

Motifs and footprints are direct and indirect indicators of regulatory events respectively. The difficulty in detecting footprints comes from both enzymatic cutting bias and weak signals from transient TFs. Instead of defining footprints with a mathematical formula, recent publications made a good first attempt to embrace the fast development of machine learning algorithms with supervised learning [131, 144]. Moreover, nucleosome detection remains difficult due to an intrinsic weakness of ATAC-seq data where low read coverage beyond peaks is typical. NucleoATAC and HMMRATAC have attempted this; however, large gaps in methods of detection remain in this area.

Another consideration for analysis is on reconstructing gene regulatory networks from ATAC-seq data alone or integrating with multiomics data. This is particularly tempting because ATAC-seq can work with as low as 500 cells and allow the study of well-defined subpopulations especially in developmental biology and clinical samples. ScATAC-seq provides another option to study chromatin biology in heterogeneous cell populations.

In summary, ATAC-seq, an information rich assay, is in great demand for specific bioinformatic analysis tools for further exploitation in analyzing chromatin state, TF footprint, nucleosome position, and regulatory network reconstruction. As a starting point, we suggest researchers can build an effective workflow, by combining FastQC, trimmomatic, and BWA-MEM for pre-analysis, and MACS2 for peak calling. For advanced analysis, we suggest csaw for differential peak analysis, MEME suite for motif detection and enrichment, ChIPseeker for annotation and visualization, HMMRATAC for nucleosome detection, and HINT-ATAC for footprint analysis. If RNA-seq data is available, regulatory networks can be reconstructed using PECA method. However, researchers can always refer to this review for alternative tools for each step and we recommend selecting the tool based on the context of the experimental system and the data collected.

We envisage that this review will encourage researchers to appreciate the complexity and current major hurdles in ATAC-seq data analysis. New ATAC-seq-specific tools and comprehensive benchmarking studies would enable the answering of more biological questions with ATAC-seq in the near future.

## Supplementary information

Additional file 1. Review history. (DOCX 21 kb)
