# Supplementary material for: From reads to insight: a hitchhiker’s guide to ATAC-seq data analysis
Source: Genome Biol. 2020 Feb 3;21:22. doi: 10.1186/s13059-020-1929-3 (PMC6996192; doi:10.1186/s13059-020-1929-3)
Supplement: Supplementary file 1 — Review history. (DOCX 21 kb) [file 13059_2020_1929_MOESM1_ESM.docx]

Review History

**First round of review**

**Reviewer 1**

**Is the topic of the article timely and of interest to a wide range of readers?**

No: Although the topic of ATAC-seq analysis is timely and interesting, I do not believe that this article provides any real insights that will interest readers of Genome Biology.

**Is the article well written and presented in a logical way?**

No: The overall organization is logical, but the writing is awkward in many places and full of minor grammatical and mechanical errors.

**Do the authors cover the relevant literature in an accurate and balanced way?**

Yes

**Do the authors provide a useful synthesis of the topic?**

No: This is one of my biggest criticisms of the review. The description of previous work feels bogged down by details and lacks the big-picture perspective that I look for in a review. The majority of the text is dedicated to describing each of many different methods and how they work, without really synthesizing or drawing any conclusions from this information. For example, it would be very helpful to provide insights about gaps in the field that future method development should address.

**Do the authors provide insightful discussion of the future directions for the field?**

No

**Do any tables, figures or boxes that are present enhance the manuscript?**

No: Only 2 figures are provided. The first figure shows a schematic representation of ATAC-seq and a couple of sample data visualizations. Figure 2 is a simple flowchart diagram that shows analysis steps, inputs, and outputs. In my opinion, neither figure does a very good job of clarifying, summarizing, or synthesizing the main points of the paper.

**Would the manuscript benefit from any additional tables, figures or boxes?**

Yes

**Comments to author:**

Yan and colleagues wrote a review article describing the steps in standard analysis of ATAC-seq data and summarizing existing methods for each step. On the whole, the paper is logically organized and this topic could be of interest to many readers. However, the writing is awkward and hard to read in many places and contains many minor grammatical errors. Even worse, the review is completely bogged down by detailed descriptions of dozens of individual computational approaches and I did not glean any deep insights from reading it. The review lacks the sort of synthesis that would provide motivation for readers to keep reading. Finally, only two figures are included, and they do not significantly clarify, summarize, or synthesize the main points of the paper.

**Reviewer 2**

**Is the topic of the article timely and of interest to a wide range of readers?**

Yes

**Is the article well written and presented in a logical way?**

Yes

**Do the authors cover the relevant literature in an accurate and balanced way?**

Yes

**Do the authors provide a useful synthesis of the topic?**

Yes

**Do the authors provide insightful discussion of the future directions for the field?**

No

**Do any tables, figures or boxes that are present enhance the manuscript?**

Yes

**Would the manuscript benefit from any additional tables, figures or boxes?**

Yes

**Comments to author:**

ATAC-seq can measure chromatin accessibility with the advantages in less-cell number and non-intensive labor. It has been applied in various biological studies. This review provides a timely and comprehensive review for the technology and analysis method.

Overall, the review is quite ambitious to covering all the topics and lacks solid materials.

The authors list many facts but no deep discussion. Instead, I suggest the authors to start from their own work in ATAC-seq data and focus on one specific direction.

The title should be revised to be more specific and focus. The current terms"insight" and "landscape" are not fully addressed with limited materials. The authors should highlight some aspects according to the possible target audience. Especially, the audience of genome biology are from the bioinformatics community. They are familiar with the pipeline and the authors should provide more advanced analysis technique.

I suggest the authors to provide a survey for the ATAC-seq data resources. For example, how many datasets are in the GEO database and how is the increasing trend compared with RNA-seq or DNaseq-seq?

I also suggest the authors to provide one example dataset and report the various results and analysis mentioned in the review.

Some very relevant literature should be cited as follows. Those include the joint modeling with RNA-seq and some deep learning based modeling.

Modeling the causal regulatory network by integrating chromatin accessibility and transcriptome data

National Science Review 3 (2), 240-251

Integrative analysis of single cell genomics data by coupled nonnegative matrix factorizations

Proc Natl Acad Sci U S A 115 (30), 7723-7728

Chromatin accessibility prediction via a hybrid deep convolutional neural network

Bioinformatics 34 (5), 732-738

Chromatin accessibility prediction via convolutional long short-term memory networks with k-mer embedding

Bioinformatics 33 (14), i92-i101

**Authors Response**

**Point-by-point responses to the reviewers’ comments:**

*We are grateful to the reviewers’ criticisms and constructive suggestions that have helped to improve our manuscript. To address the reviewers’ concerns, we have included a number of critical experiments accordingly and made extensive revision in our revised manuscript. The point-by-point responses are listed below:*

Reviewer #1: Yan and colleagues wrote a review article describing the steps in standard analysis of ATAC-seq data and summarizing existing methods for each step. On the whole, the paper is logically organized and this topic could be of interest to many readers.

However, the writing is awkward and hard to read in many places and contains many minor grammatical errors. Even worse, the review is completely bogged down by detailed descriptions of dozens of individual computational approaches and I did not glean any deep insights from reading it. The review lacks the sort of synthesis that would provide motivation for readers to keep reading.

*RESPONSE: We thank reviewer #1 for their comments. We have attempted to appraise the current landscape of ATAC-seq and demonstrate the plethora of tools available for analysis, it is essential to pay attention to the certain critical analysis steps to ensure the accuracy of the results to achieve interpretable insights. This was the main motivation of our manuscript. To clarify the narrative, we have summarized ATAC-seq into four major steps and furnished a flow diagram in figure 2 (pre-analysis, core analysis, advanced analysis and integration with multi-omics data) in our revised manuscript. We have reorganized and rewritten some paragraphs (especially pre-analysis, nucleosome position) to make it clearer to read and follow. We have native English speakers to correct the language issues. Superfluous technical details were removed, and additional synthesis of our experience was added (especially in peak calling and footprinting, highlighted in yellow), which could provide our precious tips on ATAC-seq analysis for all readers.*

Finally, only two figures are included, and they do not significantly clarify, summarize, or synthesize the main points of the paper.

*RESPONSE: We have reorganized Figure 1 and 2. In figure 2, the roadmap of ATAC-seq data analysis was reorganized and split into four major steps, with key points in each step highlighted. In figure 1 and 3, we provided sample output for several key steps, in order to provide researchers with intuitive understanding of the analysis output. In figure 4, we provided a pictorial summary of tools in peak calling and peak differential analysis steps, which we believe is more informative for readers to understand the key points. However, due to the limits of the display items from the journal, we are only able to display 4 figures and 1 table in this review.*

Reviewer #2:

ATAC-seq can measure chromatin accessibility with the advantages in less-cell number and non-intensive labor. It has been applied in various biological studies. This review provides a timely and comprehensive review for the technology and analysis method.

Overall, the review is quite ambitious to covering all the topics and lacks solid materials.

The authors list many facts but no deep discussion. Instead, I suggest the authors to start from their own work in ATAC-seq data and focus on one specific direction.

*RESPONSE: Thank you for your precious suggestions. We appraise the current tools available for analysis and have summarized ATAC-seq into four major steps and a roadmap in figure 2 (pre-analysis, core analysis, advanced analysis and integration with multi-omics data) with key points highlighted for each step in our revised manuscript. In each step, we discussed the methods available and their pros and cons in order to encourage future improvement of current tools. We think it is helpful to add some example data from our analysis of public ATAC-seq datasets in leukemia (Corces et al, Nature Genetics 2016). We updated the figures and manuscripts accordingly, which we believe, will make it easier for the readers to understand.*

The title should be revised to be more specific and focus. The current terms"insight" and "landscape" are not fully addressed with limited materials. The authors should highlight some aspects according to the possible target audience. Especially, the audience of genome biology are from the bioinformatics community. They are familiar with the pipeline and the authors should provide more advanced analysis technique.

*RESPONSE: We thank reviewer 2 for their feedback, we have changed the title to be more specific based on your suggestion and have revised our manuscript to be a guide to ATAC-seq. We believe our manuscript is helpful to the bioinformatics community in terms of highlighting the current hurdles and challenges in ATAC-sea data analysis and summarising the available tools for analysis of ATAC-seq data (there are many). We want to bring the reader’s attention to the plethora of tools available and hope this will encourage more in-depth evaluation of current tools as well as develop new ATAC-seq specific tools. Our revised flow diagram in figure two will take the reader through the critical steps for ATAC-seq analysis and we have expanded on certain points that would require consideration (which tool) to enhance the chance of interpretable insights.*

I suggest the authors to provide a survey for the ATAC-seq data resources. For example, how many datasets are in the GEO database and how is the increasing trend compared with RNA-seq or DNaseq-seq?

*RESPONSE: We have added the survey of ATAC-seq data comparing to other epigenomics data (Fig 1A). Intriguingly, the number increases almost exponentially, and exceeds DNase-seq and FAIRE-seq, which demonstrate the value of ATAC-seq in different applications and highlight the necessity and urgency of our review on the ATAC-seq data analysis. In addition, our summary table and figure for bioinformatics tools in several key steps will be a valuable resource for ATAC-seq practitioners.*

I also suggest the authors to provide one example dataset and report the various results and analysis mentioned in the review.

*RESPONSE: We added sample output for post alignment QC (Figure 1B, C), peak calling (Figure 3B), peak annotation (Figure 1D), nucleosome position and footprinting (Figure 3B). We hope these results will give more intuitive explanation of these steps.*

Some very relevant literature should be cited as follows. Those include the joint modeling with RNA-seq and some deep learning based modeling.

Modeling the causal regulatory network by integrating chromatin accessibility and transcriptome data

National Science Review 3 (2), 240-251

Integrative analysis of single cell genomics data by coupled nonnegative matrix factorizations

Proc Natl Acad Sci U S A 115 (30), 7723-7728

Chromatin accessibility prediction via a hybrid deep convolutional neural network

Bioinformatics 34 (5), 732-738

Chromatin accessibility prediction via convolutional long short-term memory networks with k-mer embedding

Bioinformatics 33 (14), i92-i101

*RESPONSE: Thank you for pointing out these great publications that we missed. I have added these four publications to our manuscript (1st and 2nd in Page 9 Line 183, 3th in Page 4 Line 70, 4th in Page 20 Line 448), which help us improve the quality of the manuscript.*

*We hope we have made the requisite revisions to our manuscript for review and consideration for publication in Genome Biology.*

*We thank you for your time and attention.*

**Second round of review**

**Reviewer 1**

I commend the authors for significantly improving the figures, which now clarify and amplify the text. I also find the simplified organization of the text (four main steps, reflected in Fig. 2) to be much more comprehensible. However, the writing is still quite problematic. I underlined around 70 grammatical errors and non-idiomatic phrasings in the text (see scanned pdf attachment), some of which make the meanings of the offending sentences completely incomprehensible. Additionally, as before, although I carefully read the entire review, I did not come away with any significant insights. The review still reads like a laundry list of methods and short, obvious statements about them. I don’t know exactly what to suggest to fix this problem. It will certainly require thinking carefully about precisely what your unique insights are. Is the point of the review to identify best practice analysis guidelines, identify suggestions for future research directions, or something else? Stating these goals clearly and then describing the insights in a way that makes them stand out from the long list of methods being summarized (perhaps in a section at the end) may help. In its current form, I don’t think many readers will persist to the end of this review. Finally, although the abstract mentions single-cell ATAC-seq (and I think this is a topic of considerable interest to readers), there is no section devoted to this topic, and only a few papers are cited. Because this topic is much more recent, it may be easier to contribute some unique insights that will interest readers.

Specific Comments:

-     The term “transcriptional regulatory network” should be replaced throughout with “transcriptional regulatory networks”, which is more conventional and reflects the fact that there is not a single network. Network inference is nearly always context-specific.

-     Some of the box labels in Fig. 2 are unclear/confusing. For example, I don’t know what “quantitative or qualitative” on the “RNA-seq” box means. Why does the Nucleosome position box say “Low depth beyond peaks”? Another odd one is “TF or histone marker” for the “ChIP-seq” box. Perhaps the problem is that the summary text means something different for each box (e.g., the way in which nucleosome position is estimated vs. the types of ChIP-seq data). Another non-parallelism is the fact that 2 of the green boxes are data types and the third is an analysis type. Making each box and summary text communicate the same type of information is essential for readers to understand the figure.

-     The label “machine learning” in Fig. 4A is not a great choice, because all of the approaches can be considered “machine learning” in some sense. Perhaps “Markov model” would be more appropriate. Furthermore, the top-level label should be “peak calling” (not "peak caller") to make it grammatically parallel to “peak differential analysis” in Fig. 4B.

-       The figure captions contain multiple grammatical errors and non-idiomatic phrasings. I did not underline these, but please correct them.

**Reviewer 2**

The authors fully considered my suggestions by adding a detailed analysis example and some supporting evidence. The logic of four steps analysis is also better to readers. The manuscript has been greatly improved. I suggest its acceptance now.

**Authors Response**

**Point-by-point responses to the reviewers’ comments:**

Reviewer #1: I commend the authors for significantly improving the figures, which now clarify and amplify the text. I also find the simplified organization of the text (four main steps, reflected in Fig. 2) to be much more comprehensible. However, the writing is still quite problematic. I underlined around 70 grammatical errors and non-idiomatic phrasings in the text (see scanned pdf attachment), some of which make the meanings of the offending sentences completely incomprehensible.
We thank reviewer #1 for the comments. We have revised these grammatical errors and fixed some non-idiomatic phrasings to make the text clear and easy to understand.

Additionally, as before, although I carefully read the entire review, I did not come away with any significant insights. The review still reads like a laundry list of methods and short, obvious statements about them. I don't know exactly what to suggest to fix this problem. It will certainly require thinking carefully about precisely what your unique insights are. Is the point of the review to identify best practice analysis guidelines, identify suggestions for future research directions, or
something else? Stating these goals clearly and then describing the insights in a way that makes them stand out from the long list of methods being summarized (perhaps in a section at the end) may help. In its current form, I don't think many readers will persist to the end of this review.
*We thank reviewer #1 for the comments. We thought it may be helpful to add a suggested pipeline for analysis as a starting point for new researchers in ATAC-seq, in order to help them to get started on the analysis leading to interpretable insights. We have added one paragraph as our suggested best practice starting point. We have also summarized this as part of figure 2 legend together with the flow diagram (see the highlights in the figure legend).
` In summary, ATAC-seq, an information rich assay, is in great demand for specific bioinformatic analysis tools for further exploitation in analysing chromatin state, TF footprint, nucleosome position and regulatory network reconstruction. As a starting point, we suggest researchers can build an effective workflow, by combining FastQC, trimmomatic and BWA-MEM for pre-analysis, and MACS2 for peak calling. For advanced analysis, we suggest csaw for differential peak analysis, MEME suite for motif detection and enrichment, ChIPseeker for annotation and visualization, HMMRATAC for nucleosome detection and HINT-ATAC for footprint analysis. If RNA-seq data is available, regulatory networks can be reconstructed using PECA method. However, researchers can always refer to this review for alternative tools for each step and we recommend selecting the tool based on the context of the experimental system and the data collected. `*

Finally, although the abstract mentions single-cell ATAC-seq (and I think this is a topic of considerable interest to readers), there is no section devoted to this topic, and only a few papers are cited. Because this topic is much more recent, it may be easier to contribute some unique insights that will interest readers.
*We thank reviewer #1 for the comments. Single cell ATAC-seq is very new and we have added some comments regarding this technique especially highlighting the clustering analysis, and the approaches to overcome data sparsity. We also moved the combined single cell chromatin accessibility assay part to this section.
`Single-cell ATAC-seq
Enabled by microfluidic, nano-well and combinatorial indexing technologies, scATAC-seq is now able to measure the chromatin accessibility for thousands of cells with easy protocol at low cost [33–35]. The chromatin accessibility at each base will be binary and the scATAC-seq data will be sparse because in diploid organisms, there are only two copies of DNA. This is a challenge in analysing scATAC-seq data. Despite the analyses listed for bulk ATAC-seq, another important analysis for single-cell is clustering. A recent benchmarking study from Chen et al. about clustering methods in scATAC-seq showed that SnapATAC, Cusanovich2018 and cisTopic outperformed other methods [23,172–174]. These three methods are featured by workflows combining window based genome binning, binarization of the accessibility, coverage bias correction and dimension reduction using principle component analysis, which specifically handle the sparse scATAC-seq data [174]. This study provides a useful insight for future scATAC-seq software development.
New techniques such as scNMT-seq, sci-CAR and Pi-ATAC were recently developed to measure chromatin accessibility, transcriptome and proteome simultaneously from exactly the same cell [160,175,176]. Data from these experiments could help to deduce the complex interplay between the epigenome, transcriptome and proteome and help us to understand why different cells behave distinctively. With the advantages of single cell analysis are clear, there are challenges. Cost and time efficient single cell techniques as well as bioinformatic tools remain an area of active research and development.`*

Specific Comments:
- The term "transcriptional regulatory network" should be replaced throughout with "transcriptional regulatory networks", which is more conventional and reflects the fact that there is not a single network. Network inference is nearly always context-specific.
*We thank reviewer #1 for the comments. We have corrected all these grammatical errors.*

- Some of the box labels in Fig. 2 are unclear/confusing. For example, I don't know what "quantitative or qualitative" on the "RNA-seq" box means. Why does the Nucleosome position box say "Low depth beyond peaks"? Another odd one is "TF or histone marker" for the "ChIP-seq" box. Perhaps the problem is that the summary text means something different for each box (e.g., the way in which nucleosome position is estimated vs. the types of ChIP-seq data). Another non-parallelism is the fact that 2 of the green boxes are data types and the third is an analysis type. Making each box and summary text communicate the same type of information is essential for readers to understand the figure.
*We thank reviewer #1 for the comments. To make it clear, we have added some explanation for figure legend of figure2.
` Text in each box emphasizes the important considerations in each analysis step. `
We also rephrased the words in the box to make it clearer. We changed qualitative and quantitative to 'Overlap DEGs with differential peaks' and 'Regression (expression ~ accessibility)'. For nucleosome positioning, we changed the text to 'All or nucleosomal fragments' and 'Low coverage beyond peaks'.
We also changed the text in the green boxes to be parallel.*

- The label "machine learning" in Fig. 4A is not a great choice, because all of the approaches can be considered "machine learning" in some sense. Perhaps "Markov model" would be more appropriate. Furthermore, the top-level label should be "peak calling" (not "peak caller") to make it grammatically parallel to "peak differential analysis" in Fig. 4B.
*We thank reviewer #1 for the comments. We have changed the text accordingly for figure 4.*

- The figure captions contain multiple grammatical errors and non-idiomatic phrasings. I did not underline these, but please correct them.
*We thank reviewer #1 for the comments. We have corrected the grammatical errors in figure legends.*
